# Detection of autoreactive CD4 T cells using major histocompatibility complex class II dextramers

**DOI:** 10.1186/1471-2172-12-40

**Published:** 2011-07-18

**Authors:** Chandirasegaran Massilamany, Bijaya Upadhyaya, Arunakumar Gangaplara, Charles Kuszynski, Jay Reddy

**Affiliations:** 1 Department of Pathology and Microbiology, University of Nebraska Medical Center, Omaha, NE 68198, USA; 2 Nebraska Center for Virology, University of Nebraska-Lincoln, Lincoln, NE 68583, USA; 3 School of Veterinary Medicine and Biomedical Sciences, University of Nebraska-Lincoln, Lincoln, NE 68583, USA

**Keywords:** Antigen-specific CD4 cells, Central nervous system, Dextramers, Experimental autoimmune encephalomyelitis, Experimental autoimmune myocarditis, Heart, Major histocompatibility complex class II, Myelin oligodendrocyte glycoprotein, Cardiac myosin heavy chain-alpha, Proteolipid protein, Tetramers

## Abstract

**Background:**

Tetramers are useful tools to enumerate the frequencies of antigen-specific T cells. However, unlike CD8 T cells, CD4 T cells - especially self-reactive cells - are challenging to detect with major histocompatibility complex (MHC) class II tetramers because of low frequencies and low affinities of their T cell receptors to MHC-peptide complexes. Here, we report the use of fluorescent multimers, designated MHC dextramers that contain a large number of peptide-MHC complexes per reagent.

**Results:**

The utility of MHC dextramers was evaluated in three autoimmune disease models: 1) proteolipid protein (PLP) 139-151-induced experimental autoimmune encephalomyelitis in SJL/J (H-2^s^) mice; 2) myelin oligodendrocyte glycoprotein (MOG) 35-55-induced experimental autoimmune encephalomyelitis in C57Bl/6 (H-2^b^) mice; and 3) cardiac myosin heavy chain (Myhc)-α 334-352-induced experimental autoimmune myocarditis in A/J (H-2^a^) mice. Flow cytometrically, we demonstrate that IA^s^/PLP 139-151, IA^b^/MOG 35-55 and IA^k^/Myhc-α 334-352 dextramers detect the antigen-sensitized cells with specificity, and with a detection sensitivity significantly higher than that achieved with conventional tetramers. Furthermore, we show that binding of dextramers, but not tetramers, is less dependent on the activation status of cells, permitting enumeration of antigen-specific cells *ex vivo*.

**Conclusions:**

The data suggest that MHC dextramers are useful tools to track the generation and functionalities of self-reactive CD4 cells in various experimental systems.

## Background

Traditionally, limiting dilution analysis and cytokine ELISPOT assays have been used to enumerate the frequencies of antigen-specific cells, but low specificity and the tedious nature of these assays limit their use for routine applications [1-3]. To overcome these limitations, tetramer technology has been developed [4]. This approach involves derivation of fluorescent dye-labeled tetramerized complexes containing peptide-assembled major histocompatibility complex (MHC) molecules. The use of tetramers permits easy and rapid detection of antigen-sensitized T cells at a single cell level by flow cytometry (FC) [4,5]. Additionally, tetramer reagents can be used in conjunction with antibodies to phenotypically characterize the generation and expansion of antigen-specific cells during ensuing immune responses and to study their functionalities by cell sorting.

Unlike with MHC class I tetramers, direct enumeration of antigen-specific cells - in particular, rare and low-affinity autoreactive CD4 T cells - with MHC class II tetramers has been difficult. Under natural conditions, interaction between T cells and antigen-presenting cells involves engagement of MHC molecules with multiple T cell receptors (TCRs), but their binding strength is low. Hence, their stability requires interaction between various adhesion molecules. Tetramers are artificially created peptide-assembled MHC complexes, and their interactions with TCRs occur in the absence of accessory molecules, a limitation that exists with both class I and class II tetramers. In spite of this limitation, class I tetramers can generally bind CD8 T cells, but require participation of a CD8 coreceptor [6]. By comparison, participation of a CD4 coreceptor is not critical for class II tetramers to bind to CD4 cells [7,8]. This differential requirement of coreceptors may, in part, explain differences in the abilities of tetramers to bind respective T cell populations.

It is generally accepted that class II tetramers preferentially bind activated CD4 cells expressing high amounts of CD4 and CD25 [9-12]. We and others had previously proposed that activation might result in reorientation and configuration of TCR and accessory molecules, leading to enhanced avidity of TCR-MHC binding [11,13,14], thus restricting the utility of the reagents for direct enumeration of the frequencies of antigen-sensitized cells *ex vivo*. Recently, MHC dextramers bearing dextran backbones assembled with peptide-tethered MHC class I molecules have been shown to detect antigen-specific CD8 T cells [15,16]. Our studies involve the use of a streptavidin (SA)-dextramer conjugate of 270 kDa, which has been validated to detect tumor- and virus-specific cells in various systems [15,17-19]. To address the utility of dextramers, we used three autoimmune disease models: 1) proteolipid protein (PLP) 139-151-induced experimental autoimmune encephalomyelitis (EAE) in SJL/J (H-2^s^) mice; 2) myelin oligodendrocyte glycoprotein (MOG) 35-55-induced EAE in C57Bl/6 (H-2^b^) mice; and 3) cardiac myosin heavy chain (Myhc)-α 334-352-induced experimental autoimmune myocarditis (EAM) in A/J (H-2^a^) mice. We demonstrated that MHC class II dextramers detect self-reactive CD4 T cells with specificity and to a greater sensitivity than conventional tetramers. We showed that binding of dextramers, but not tetramers, is less dependent on activation status of the cells, thus permitting the reagents to determine the frequencies of antigen-specific cells *ex vivo *in immunized mice.

## Methods

### Mice

Four-to-six-week-old female SJL/J (H-2^s^) and C57Bl/6 (H-2^b^) mice and six-to-eight-week-old A/J (H-2^a^) mice were procured from the Jackson Laboratory (Bar Harbor, ME). The mice were maintained according to the animal protocol guidelines of the University of Nebraska-Lincoln, Lincoln, NE.

### Peptide synthesis and immunization procedures

PLP 139-151 (HSLGKWLGHPDKF), MOG 35-55 (MEVGWYRSPFSRVVHLYRNGK) and Myhc-α 334-352 (DSAFDVLSFTAEEKAGVYK) were synthesized on 9-fluorenylmethyloxycarbonyl chemistry (Neopeptide, Cambridge, MA). All peptides were HPLC-purified (>90%), identity was confirmed by mass spectroscopy, and the peptides were dissolved in 1 × PBS prior to use. To generate primary T cell cultures for PLP 139-151 and MOG 35-55, SJL and C57Bl/6 mice, respectively, were immunized with the above peptides emulsified in complete Freund's adjuvant (CFA) subcutaneously (s.c.) in multiple sites in the flank and sternal regions (100 μg/mouse) [5,20]. For immunization of A/J mice with Myhc-α 334-352, the peptide was emulsified in CFA supplemented with *Mycobacterium tuberculosis *(M. tb; Difco Laboratories, Detroit, MI) extract to a final concentration of 5 mg/ml and injected s.c. in the flank, foot pads and sternal regions (100 μg/mouse). For disease induction, however, regardless of peptides used, emulsions were prepared in CFA containing M. tb extract to a final concentration of 5 mg/ml. To induce EAE with PLP 139-151 or MOG 35-55, peptide emulsions were given as a single dose [11,20], whereas for EAM induction, two doses of emulsions containing Myhc-α 334-352 were administered with an interval of one week [21,22]. Nonetheless, all animals involved in the disease-induced protocols received pertussis toxin (List Biological Laboratories, Campbell, CA) intraperitoneally on day 0 and day 2 after the first immunization (PLP 139-151 and Myhc-α 334-352, 100 ng/mouse; MOG 35-55, 200 ng/mouse) [5,20,21]. SJL and C57Bl/6 mice were monitored for the appearance of clinical signs of EAE and scored as described [23,24]: 0 - healthy; 1 - limp tail or hind limb weakness but not both; 2 - limp tail and hind limb weakness; 3 - partial paralysis of hind limbs; 4 - complete paralysis of hind limbs; 5 - moribund or dead.

### Generation of antigen-specific primary T cell cultures

Ten days after immunizations with peptides, mice were sacrificed, and the maxillary, mandibular, axillary, inguinal and popliteal draining lymph nodes (LN) were collected. Single cell suspensions were prepared after lysing the erythrocytes with 1 × ammonium chloride potassium buffer (Lonza, Walkersville, MD). Lymph node cells (LNC) were stimulated with the peptides (PLP 139-151, 20 μg/ml; MOG 35-55 and Myhc-α 334-352, 50 μg/ml) at a density of 5 × 10^6 ^cells/ml for two days in RPMI medium supplemented with 10% fetal bovine serum (FBS), 1 mM sodium pyruvate, 4 mM L-glutamine, 1 × each of non-essential amino acids and vitamin mixture, 100 U/ml penicillin-streptomycin and 50 μg/ml gentamicin (Lonza). After two days, cultures were supplemented with the above medium containing interleukin (IL)-2 (eBioscience, San Diego, CA), hereafter termed growth medium. Viable lymphoblasts were harvested two to three days later by Ficoll-Hypaque density gradient centrifugation, and the cultures were maintained in growth medium.

### Isolation of mononuclear cells (MNC) from CNS tissues and hearts

After euthanization, mice were perfused by injecting 10 ml of 1 × cold PBS into the left ventricle of the hearts; brains were harvested by blunt dissection; and spinal cords were flushed out using 1 × cold PBS. The tissues were homogenized and digested with type IV collagenase (400 U/ml; Worthington, Lakewood, NJ) at 37°C for 1 hour, and MNC were harvested by percoll density gradient centrifugation (70%/30%) as described previously [25]. To harvest MNC from hearts, animals were first perfused as above, and hearts were collected and minced. The tissue suspensions were then incubated with a buffer containing 1 × PBS, collagenase and 2% FBS at 37°C for 15 min in a shaker with continuous agitation. The digested tissues were passed through an 18G needle and incubated for an additional 10 minutes; the cell pellets were obtained by centrifugation and resuspended in 40% buffered percoll. Cell suspensions were overlaid with 75% percoll and centrifuged; the interphase representing MNC was then collected and washed [26,27].

### Derivation of IA^s^, IA^b ^and IA^k ^tetramers and dextramers

IA^s^/PLP 139-151 and IA^s^/Theiler's murine encephalomyelitis virus (TMEV) viral capsid protein_2 _70-86, IA^b^/MOG 35-55, and IA^k^/Myhc-α 334-352 and IA^k^/Bovine ribonuclease (RNase) 43-56 tetramers were generated as previously described [5,11,25,28]. While we generated the IA^k ^constructs [11,29], the IA^s ^and IA^b ^constructs were kindly gifted by Dr. Vijay Kuchroo, Harvard University, Boston, MA. Briefly, α and β constructs for each IA allele containing the sequences of the respective peptides were expressed in a Baculovirus system using Sf9 insect cells (Invitrogen, Carlsbad, CA) and soluble MHC molecules were obtained [5,11]. IA^s^, IA^b ^and IA^k ^monomers were purified on antibody columns prepared using MKS4, M5114, and 10-2.16 (Bio × Cell, West Lebanon, NH), respectively and the protein yield generally ranged from 0.5 mg to 1 mg/L [5,11,25,28]. After concentrating, the soluble MHC proteins were biotinylated using biotin protein ligase at an optimized concentration of 25 μg/10 nmol of substrate as recommended by the manufacturer (Avidity, Denver, CO). The biotinylated proteins were then incubated with SA conjugated with a fluorescent dye - fluorescein isothiocyanate (FITC), phycoerythrin (PE) or allophycocyanin (APC) - at a 4:1 ratio for one hour on ice. The reagents thus prepared are referred to as tetramers. To prepare dextramers, biotinylated soluble monomers of all three IA alleles (IA^s^, IA^b ^and IA^k^) containing the peptides were coupled to activated dextran backbones (kindly provided by Immudex Aps, Copenhagen, Denmark) at various molar ratios in 1 × Tris Hcl 0.05 M, pH 7.2 for 30 minutes at room temperature (RT) and the preparation of fluorochrome-labeled dextran backbone has been previously described [16]. Fluorochrome-labeled dextramers were prepared by mixing biotinylated IA monomers with dextran molecules. For example, dextramer-PE reagents were prepared by mixing 1.6 × 10^-11 ^moles of dextran-PE molecules with 3.17 × 10^-10 ^moles of IA^s ^monomers which resulted a molar ratio of 1:20. All the reagents were aliquoted and stored at -80°C or 4°C until further use.

### Tetramer or dextramer staining

#### (a) Cells activated in vitro

To enumerate the frequencies of tetramer (tet^+^) or dextramer (dext^+^) cells, viable lymphoblasts harvested from cultures prepared from immunized SJL, C57Bl/6 or A/J mice were used for tet or dext staining as described previously [5,30]. Briefly, cells were stained with tetramers or dextramers in growth medium, pH 7.6, containing 2.5% FBS at RT for up to 3 hours, followed by staining with anti-CD4 (eBioscience, San Diego, CA) and 7-aminoactinomycin D (7-AAD; Invitrogen). After washing, cells were analyzed by flow cytometry (FC; FACSCalibur, BD Biosciences, San Diego, CA). Percentages of tet^+ ^or dext^+ ^cells were then determined in the live (7-AAD^-^) CD4 subset using Flow Jo software (Tree Star, Ashland, OR). In some experiments, antigen-sensitized cells were rested for two weeks in growth medium and treated with or without neuraminidase (NASE) (0.7 U/ml) (Type X from *Clostridium perfringens*; Sigma-Aldrich, St. Louis, MO) prior to tet or dext staining [11].

#### (b) Ex vivo staining

LNC were obtained from a pool of draining LN harvested from groups of mice immunized with various peptides. CD4 cells were enriched from LNC to a purity of more than 95% by negative selection based on magnetic separation using IMAG as recommended by the manufacturer (BD Biosciences). After treating the cells with or without NASE as above, cells were washed in 5 ml of 1 × PBS; incubated with tetramers or dextramers for two hours at RT in growth medium, pH 7.6, containing FBS (2.5%). Cells were washed twice with 5 ml of 1 × PBS and stained with anti-CD4, anti-CD25, anti-CD44 (all from eBioscience) and 7-AAD and analyzed by FC. The percentages of tet^+ ^or dext^+ ^cells were determined in the live (7-AAD^-^) cells corresponding to CD4, CD25 and CD44 subsets. To stain MNC obtained from CNS tissues, cells were incubated with tetramers or dextramers directly without NASE treatment, and the percentages of tet^+ ^or dext^+ ^cells were determined as described above.

### Sorting of tet^+ ^or dext^+ ^cells by flow cytometry

LNC isolated from immunized mice were stimulated with PLP 139-151 at a density of 5 × 10^6 ^cells/ml and viable lymphoblasts were harvested on day 6 poststimulation and stained with tetramers or dextramers for two hours at RT. After washing, cells were stained with anti-CD4 and 7-AAD; the tet^-^CD4^+ ^and dext^-^CD4^+ ^cells were sorted by FC (FACSAria, BD Biosciences, San Jose, CA), and each fraction was divided into two aliquots. One aliquot of cells was immediately stained with APC-labeled PLP 139-151 or control dextramers for two hours, followed by 7-AAD. After acquiring the data by FC, dext^+ ^cells were analyzed in the live (7-AAD^-^) subset. The second aliquot (0.75 × 10^6 ^cells/ml) was rested in growth medium for two weeks and stimulated with PLP 139-151 (10 μg/ml) in the presence of irradiated antigen-presenting cells for two days; growth medium was then supplemented. Viable lymphoblasts were harvested two days later, and on day 6 poststimulation, PLP 139-151 or control tet or dext staining was performed followed by anti-CD4 and 7-AAD and the percentages of live tet^+ ^or dext^+ ^cells were enumerated as above.

### Statistics

Differences between specific and control tet or dext and also between the groups were analyzed by student's t. P ≤ 0.05 values were considered significant.

## Results

### Optimization of reaction conditions for dextramer staining

**Figure 1:**
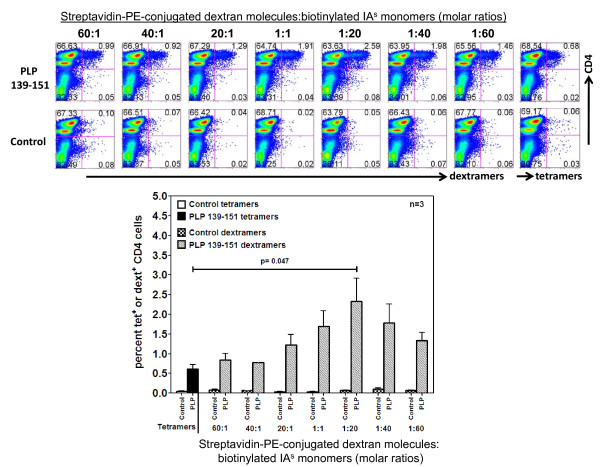
**Specificity and sensitivity of IA^s^/PLP 139-151 dextramers**. PLP 139-151 and control dextramers were prepared at the indicated molar ratios by mixing SA-conjugated dextran-PE molecules with biotinylated soluble IA^s ^molecules containing the peptide sequence for PLP 139-151. The amount of IA^s ^protein used in each of the indicated molar ratios was as follows: 60:1, 40:1, 20:1, 1:1 and 1:20, 0.43 μg; 1:40, 0.86 μg and 1:60, 1.29 μg. To prepare tetramers, 0.9 μg of IA^s ^protein containing PLP 139-151 or TMEV 70-86 was used. PLP-reactive cells were generated from SJL mice immunized with PLP 139-151, and viable cells harvested on day 6 poststimulation were stained with dextramers or tetramers followed by anti-CD4 and 7-AAD. After acquiring the cells by FC, percentages of dext^+ ^or tet^+ ^cells were enumerated in the live (7-AAD^-^) CD4 subset (top panel). In each FC plots, the upper left quadrants indicate CD4^+ ^cells containing CD4^high ^(upper) and CD4^low ^(lower) subsets. The mean ± SEM values representing three individual experiments are shown (bottom panel). Control, TMEV 70-86.

**Figure 2:**
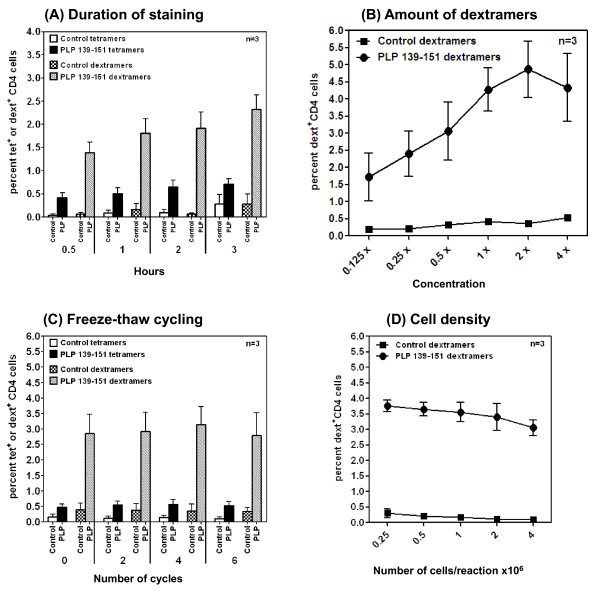
**Optimization of reaction conditions for IA^s^/PLP 139-151 dextramer staining**. **(A)** Duration of staining. PLP139-151-reactive cells generated from immunized mice were stained with PE-labeled tetramers or dextramers for PLP 139-151 and their controls. After incubating for indicated time-points, cells were stained with anti-CD4 and 7-AAD and acquired by FC; percentages of dext^+ ^or tet^+ ^cells were determined in the live (7-AAD^-^) CD4 subset. **(B)** Amount of dextramers. PLP 139-151-reactive cells were stained with the indicated concentrations of PLP 139-151 or control dextramers, and percentages of live dext^+ ^cells were determined in CD4 subset as above. The amount of biotinylated IA^s ^protein present in the 4 x-concentration was 1.72 μg and the protein concentration was halved serially in the remaining dilutions as shown. **(C)** Freeze-thaw cycling. PLP 139-151 or control tetramers and dextramers were thawed upto six times after freezing at -80°C for a minimum of half an hour between cycles and the reagents were used to stain PLP-reactive cells as above. Percentages of tet^+ ^or dext^+ ^cells were evaluated in the live CD4 subset. Control, TMEV 70-86. **(D)** Cell density. PLP 139-151 or control dextramers were used to stain PLP-reactive cells at the indicated cell numbers as above, and the percentages of dext^+ ^cells were evaluated in the live CD4 subset. Mean ± SEM values obtained from three individual experiments each involving two mice are shown.

We optimized reaction conditions for dext staining using IA^s^/PLP 139-151 dextramers, and in all reactions, TMEV 70-86 dextramers were used as controls. Dextramers were prepared using a PE-conjugated dextran polymer backbone carrying SA moieties. Each SA moiety can bind three biotinylated peptide-tethered IA^s ^molecules, whereas the fourth moiety appears not available for binding due to steric hindrance [16]. The mixture of both yielded a molar ratio of 1:20 between dextrans and IA^s ^monomers. For optimization, we prepared a series of molar ratios ranging from 60:1 to 1:60 and used them to stain PLP 139-151-reactive T cells by FC (Figure 1). The staining intensity obtained with dextramers was then compared with that of PLP 139-151 tetramers, and all reactions were performed in growth medium at RT for three hours. Figure 1 shows that PLP 139-151 dextramers prepared at all ratios stained PLP-reactive cells, and the staining was specific, since TMEV 70-86 dextramers did not show any non-specific binding. Expectedly, stimulation with PLP 139-151 resulted in two subsets of CD4 cells namely CD4^high ^and CD4^low ^and elevated CD4 expression identifies an activated and proliferating subset of cells [10,12]. We and others had previously shown that tetramers bind preferentially CD4^high ^subset [9-12]. Similar to tetramer staining, we also noted that dextramers bind CD4^high ^subset suggesting that both tetramers and dextramers stain the same subset of CD4 cells in which antigen-sensitized cells are enriched (Figure 1). The peak binding of dextramer occurred at a molar ratio of 1:20, at which dextramers detected an approximately 4-fold higher number of PLP-reactive cells than did tetramers (2.59% vs. 0.68%; p = 0.047). Therefore, we chose 1:20 molar ratio as the optimal concentration for dext staining. Importantly, by estimating the amount of protein required to make tetramers or dextramers, we noted that 1:20 molar ratio of dext preparation required 0.43 μg of IA^s ^protein per reaction, as opposed to 0.9 μg of protein required for tetramers, an ~2-fold difference. By evaluating the duration required to achieve maximum staining intensity, we noted that dextramers detected ~2- to 3-fold higher numbers of cells than did tetramers at all time-points tested (0.5 to 3 hours) (Figure 2A). The mid time-point of 2 hours was chosen for subsequent experiments since the staining intensity had nearly plateaued by then for both dextramers and tetramers. Additionally, by titrating the amount of dextramers required per reaction, we chose a dose of 1:20 molar ratio (1x) for subsequent analyses to conserve the reagent, although the staining intensity obtained by doubling the amount of dextramers tended to be high (Figure 2B). We then asked whether repeated freezing and thawing would affect dextramer binding. To address this question, we repeatedly froze and thawed tetramers and dextramers up to six cycles by freezing the reagents intermittently for a minimum duration of 30 minutes at -80°C. These reagents were used to stain PLP-reactive cells and, as shown in Figure 2C, even though the staining intensity was maintained for both dextramers and tetramers suggesting their tolerance to temperature fluctuations during storage, dextramers consistently detected higher number of antigen-specific cells than with tetramers regardless of freeze-thaw cycle number (~3.5% vs. 0.5%). Likewise, by optimizing the cell numbers required for dext staining, we noted that reactions containing cells ranging in number from 0.25 × 10^6 ^to 2 × 10^6 ^resulted in comparable binding, but thereafter, the staining intensity tended to be reduced (Figure 2D). Taken together, the data suggest that MHC dextramers detect a significantly higher proportion of antigen-reactive cells with specificity; their derivation requires less IA^s ^starting material; and the staining can be achieved in shorter time intervals than tetramers.

### FITC-labeled dextramers but not tetramers detect antigen-reactive cells

**Figure 3:**
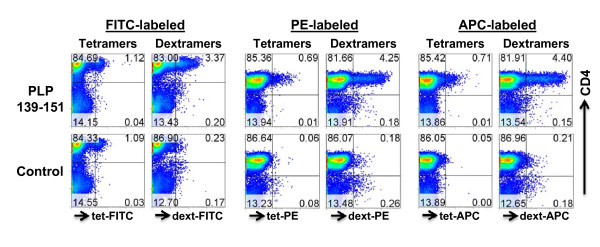
**IA^s^/PLP 139-151 dextramers, but not tetramers, labeled with FITC are useful to detect antigen-specific cells**. PLP-reactive cells generated from SJL mice immunized with PLP 139-151 were stained with PLP 139-151 or control tetramers and dextramers, each labeled with FITC, PE or APC, followed by anti-CD4 and 7-AAD. After acquiring the cells by FC, percentages of tet^+ ^or dext^+ ^cells were analyzed in the live (7-AAD^-^) CD4 subset. Data from one of the two independent experiments each involving 2 mice are shown.

Among various fluorescent dyes, fluorescent signals generated from FITC are weak and hence FITC-labeled tetramers are not generally used. Given the fact that dextramers contain more FITC moieties, we reasoned that fluoresceinated dextramers might give signals strong enough to detect antigen-reactive cells by FC. We examined this possibility by staining PLP-reactive cells with FITC-labeled PLP 139-151 or control tetramers and dextramers, and the percentages of cells positive for each were analyzed (Figure 3). Additionally, their fluorescent intensities were compared with tetramers or dextramers labeled with PE or APC. Expectedly, FITC-labeled PLP 139-151 tetramers did not yield any discernable signals that were indicative of their specificity when compared with control (TMEV) tetramers (1.12% vs. 1.09%; Figure 3, left panel). On the contrary, PLP 139-151 dextramers labeled with FITC stained a population of PLP-reactive CD4 cells (3.37%), and the cells bound with control dextramers were negligible (0.23%) proving that FITC-labeled dextramers could be used to detect antigen-specific cells. Comparison of the staining intensities of dextramers and tetramers labeled with PE and APC revealed that PE- and APC-labeled dextramers detected cell numbers ~six-fold higher than those detected with tetramers (Figure 3, right panel). Importantly, although marginally low, percentages of cells positive for FITC-labeled dextramers (3.37%) were comparable to those obtained with PE- (4.25%) and APC-(4.40%) labeled dextramers (Figure 3). The data demonstrate that, FITC can be used as a second-choice fluorescent label next to other brightly fluoresced dyes such as PE or APC to prepare dextramer reagents.

### In spite of high sensitivity, dextramers fail to detect all antigen-reactive cells

**Figure 4:**
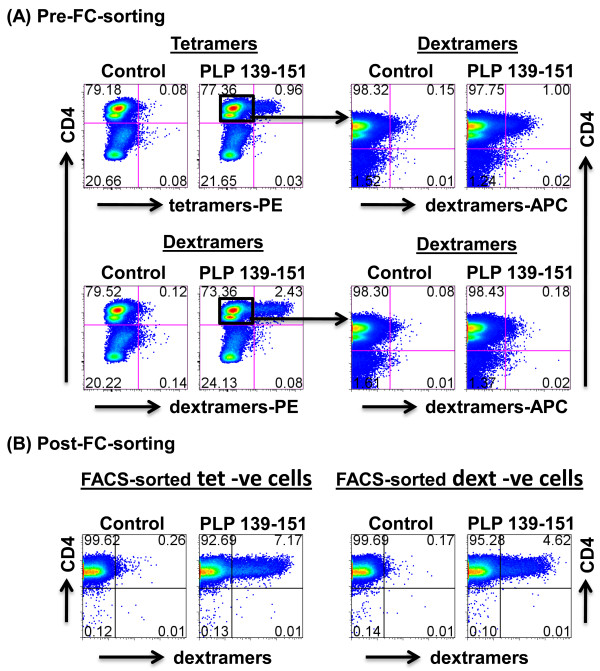
**In spite of high sensitivity, not all antigen-specific cells are detected by IA^s^/PLP 139-151 dextramers**. **(A)** Pre-FC-sorting. PLP-reactive cells generated from SJL mice immunized with PLP 139-151 were stained with PLP 139-151 or control tetramers or dextramers, followed by anti-CD4 and 7-AAD. After analyzing the cells as negative or positive for tetramers and dextramers, tet^- ^and dext^- ^CD4 cells were sorted by FC, and the cells were divided into two aliquots. One of these was immediately stained with PLP 139-151 and control dextramers, and percentages of dext^+ ^cells were evaluated in the live (7-AAD^-^) subset. **(B)** Post-FC-sorting. The second aliquot of tet^- ^and dext^- ^cells was rested for two weeks and the cells were stimulated with PLP 139-151 in the presence of irradiated antigen-presenting cells; after two days, growth medium was supplemented. Viable cells were harvested on day 6 poststimulation and stained with PLP 139-151 or control dextramers and anti-CD4 and 7-AAD. After analyzing the cells by FC, dext^+ ^and dext^- ^cells were enumerated in the live CD4 subset. Control, TMEV 70-86. Data from one of the two individual experiments each involving three mice are shown.

One of the issues being debated with tetramers is whether they can detect all of the antigen-reactive cells [14]. Since the staining intensity obtained with PLP 139-151 dextramers was higher than that obtained with tetramers, we hypothesized that dextramers can potentially bind all antigen-specific cells. To address this hypothesis, we stained PLP-reactive cells with dextramers and tetramers, and they detected ~2.4% and ~1% of cells, respectively (Figure 4A). We next sorted PLP dext^- ^and PLP tet^- ^populations by FC and divided these into two fractions. One fraction was stained with PLP 139-151 or control dextramers immediately after sorting, and frequencies were analyzed. Expectedly, PLP dextramers stained 1% of PLP tet^- ^cells and, together with the proportion of cells originally stained with tetramers (1%), constituted approximately the same number of cells (2%) obtained with dextramers (~2.4%; Figure 4A). The other fraction was rested in IL-2 medium, and two weeks later, cells were stimulated with PLP 139-151 (10 μg/ml) in the presence of syngeneic antigen-presenting cells from SJL mice. Six days poststimulation, viable lymphoblasts were stained with PLP or TMEV dextramers, and their frequencies were determined. Contrary to expectations, PLP dextramers stained cells derived from both PLP tet^- ^and PLP dext^- ^populations, but the number of dext^+ ^cells was approximately 1.5-fold higher in the PLP tet^- ^subset than in the PLP dext^- ^subset (Figure 4B). The data indicate that dextramers detect antigen-specific cells to a significantly higher proportion than the conventional tetramers, but a fraction of antigen-reactive cells can still escape from their detection.

### Activation dependency is less stringent for dextramer binding

**Figure 5:**
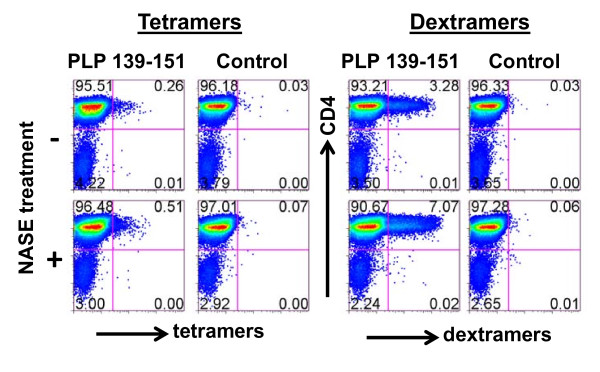
**IA^s^/PLP 139-151 dextramers bind rested antigen-specific cells better than tetramers**. PLP-reactive cells were generated from SJL mice immunized with PLP 139-151, and were rested in growth medium for 10 days. Viable cells were harvested and stained with PLP 139-151 tetramers or dextramers after being treated with or without NASE. After washing, cells were stained with anti-CD4 and 7-AAD and acquired by FC. Percentages of tet^+ ^or dext^+ ^live (7-AAD^-^) CD4 cells were then enumerated.

A major limitation of tetramers is their activation dependency in binding to T cells. We and others had previously shown that treating the cells with anti-TCR β or NASE prior to tet staining can enhance their binding by activating the cells [11,31,32]. We and others had earlier proposed that NASE might enhance adhesiveness of tetramers to the TCRs either by removing sialic acid from the cell surface, thereby reducing the net charge [31,32], or by nonspecifically activating the cells [11,32]. To address whether the use of dextramers can overcome the above limitation, we compared the staining characteristics of tetramers and dextramers using PLP-reactive cells generated from SJL mice immunized with PLP 139-151. Briefly, LNC obtained from immunized mice were stimulated with PLP 139-151 and rested in IL-2 medium. Ten days later, cells were treated with or without NASE followed by staining with tetramers or dextramers. Predictably, only 0.26% of cells treated without NASE were stained with tetramers, while 3.28% of cells were stained with dextramers, an ~12-fold difference (Figure 5). The staining was specific because TMEV tet- or TMEV dext-bound cells were negligible. NASE treatment, however, enhanced the staining intensity by ~2-fold, regardless of reagents used (tetramers: 0.26% vs. 0.51%; dextramers: 3.28% vs. 7.07%). The results suggest that dextramers can bind rested antigen-sensitized cells, and their detection sensitivity is higher than that was achieved with tetramers.

### Dextramers are useful tools to enumerate the frequencies of antigen-specific cells *ex vivo*

**Figure 6:**
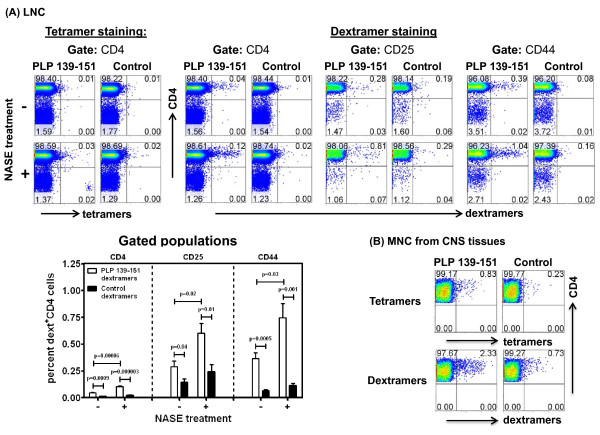
**IA^s^/PLP 139-151 dextramers detect PLP-specific cells *ex vivo***. **(A)** LNC. Groups of SJL mice were immunized with PLP 139-151 and killed 10 days later; the draining LN were harvested to prepare single cell suspensions. CD4 cells were enriched from LNC by negative selection based on magnetic bead separation. The cells were treated with or without NASE, followed by staining with PLP 139-151 or control tetramers or dextramers, followed by a cocktail containing anti-CD4 and 7-AAD and anti-CD25 or anti-CD44. After tet^+ ^and dext^+ ^live cells (7-AAD^-^) cells were acquired by FC, they were analyzed with respect to CD4, CD25 or CD44 subsets. Top panel shows representation of flow cytometric analysis, whereas the lower left panel indicates mean ± SEM values obtained from five individual experiments each involving two mice. **(B)** MNC from CNS tissues. Groups of three mice were immunized with PLP 139-151 in CFA to induce EAE, and animals showing paralytic signs were killed to harvest brains and spinal cords. The tissues were processed to obtain MNC by percoll density-gradient separation, and the cells were stained with PLP 139-151 or control tetramers or dextramers and anti-CD4 and 7-AAD. After acquiring the cells by FC, percentages of tet^+ ^and dext^+ ^cells were determined in the live CD4 population. Representative data from two individual experiments, each involving 2 to 3 mice, are shown. Control, TMEV 70-86.

After optimizing the conditions for dext staining, we sought to evaluate the utility of using dextramers to enumerate the frequencies of antigen-specific cells *ex vivo*. CD4 cells enriched from immunized SJL mice were treated with or without NASE prior to tet or dext staining, and the percentages of tet^+ ^or dext^+ ^cells were determined in CD4, CD25 or CD44 subsets, where CD25 and CD44 represent activation and memory T cell markers, respectively. Figure 6A (top left panel) demonstrates that PLP 139-151 tetramers detected a minute fraction of PLP-reactive CD4 cells in the absence of NASE treatment, and detection was barely improved with NASE treatment (0.01% vs. 0.03%). On the contrary, PLP 139-151 dextramers convincingly stained a proportion of cells expressing CD4, CD25 and CD44, even without NASE treatment. By treating the cells with NASE, the staining intensity could be increased by approximately 2-fold (CD4: 0.04% vs. 0.12%; CD25: 0.28% vs. 0.81%; CD44: 0.39% vs. 1.04%; Figure 6A, top right panel). In all these analyses, the antigen-specificity of PLP dextramers was confirmed using control dextramers (TMEV 70-86), whose binding was negligible. We then asked whether dextramers can be used to stain PLP 139-151-reactive effector cells that infiltrate CNS tissues. MNC were harvested from brain and spinal cords from mice showing partial hind limb paralysis, which had scored 3 on day 12 postimmunization. The cells were stained with tetramers or dextramers directly with no NASE treatment, and the results were compared (Figure 6B). The data revealed that while both tetramers and dextramers stain the cells with specificity, the proportion of cells stained with dextramers (2.17 ± 0.16) was ~3-fold higher than the proportion obtained with tetramers (0.74 ± 0.09; p = 0.02) and the background staining for their corresponding controls was low (0.56 ± 0.18 vs. 0.17 ± 0.07; p = 0.17). Overall, the data indicated that the use of dextramers allows us to enumerate the PLP-specific cells *ex vivo*, but their detection can be enhanced with NASE treatment.

**Figure 7:**
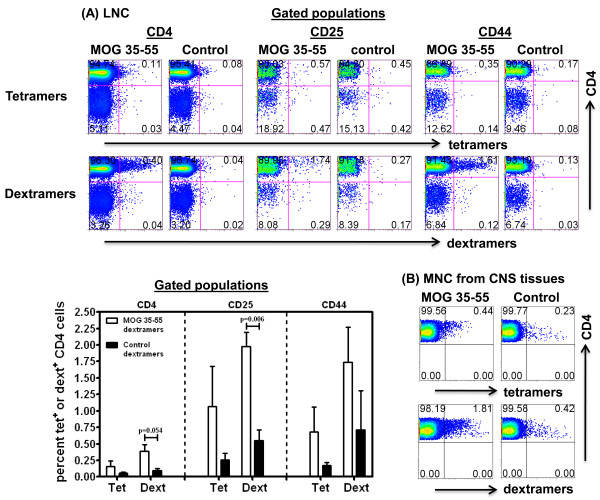
***Ex vivo *detection of MOG-reactive cells by IA**^**b**^**/MOG 35-55 tetramers and dextramers**. **(A)** LNC. CD4 cells were isolated from LNC obtained from mice immunized with MOG 35-55; after treatment with NASE, cells were stained with MOG 35-55 or control tetramers or dextramers, followed by staining with anti-CD4 and 7-AAD and anti-CD25 or anti-CD44. Cells were acquired by FC, and percentages of tet^+ ^and dext^+ ^live (7-AAD^-^) cells were analyzed in CD4, CD25 and CD44 subsets. Upper and bottom left panels, respectively, represent FC plots and mean ± SEM values obtained from three experiments each involving 3 to 5 mice. **(B) **MNC from CNS tissues. Groups of mice were immunized with MOG 35-55 in CFA, and animals showing paralytic signs were killed and brain and spinal cords collected to harvest MNC by percoll density gradient centrifugation. Cells were stained with MOG 35-55 or control tetramers and dextramers and anti-CD4 and 7-AAD. Cells were acquired by FC and tet^+ ^and dext^+ ^cells were enumerated in the live CD4 subset. Representative data from three individual experiments, each involving 3 to 5 mice, are shown. Control, PLP 139-151.

**Figure 8:**
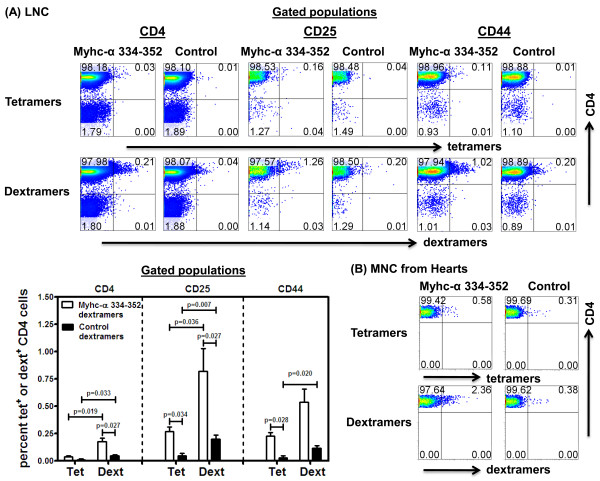
***Ex vivo *detection of Myhc-α-reactive cells by IA^k^/Myhc-α 334-352 tetramers and dextramers**. **(A) **LNC. CD4 cells were isolated from LNC harvested from immunized A/J mice and, after treatment with NASE, cells were stained with Myhc-α 334-352 or control tetramers and dextramers, followed by anti-CD4 and 7-AAD and anti-CD25 or anti-CD44. After acquiring the cells by FC, percentages of tet^+ ^and dext^+ ^live (7-AAD^-^) cells were determined in CD4, CD25 and CD44 subsets. Flow cytometric plots and mean ± SEM values obtained from four individual experiments, each involving 3 to 5 mice, are depicted in top and bottom left panels, respectively. **(B)** MNC from hearts. Groups of A/J mice were immunized with Myhc-α 334-352 and 21 days later, mice were sacrificed and hearts were collected to harvest MNC by percoll density gradient centrifugation. After staining the cells with Myhc-α 334-352 or control tetramers or dextramers and anti-CD4 and 7-AAD, cells were acquired by FC, and the percentages of tet^+ ^and dext^+ ^cells were enumerated in the live CD4 subset. Representative data from two individual experiments each involving 7 mice, are shown. Control, RNase 43-56.

We next determined the frequencies of antigen-sensitized CD4 cells in two other disease models: MOG 35-55-induced EAE in C57Bl/6 mice and Myhc-α 334-352-induced EAM in A/J mice. We created tetramers and dextramers for IA^b^/MOG 35-55 and IA^k^/Myhc-α 334-352. First, we verified that the reagents stained antigen-sensitized cells with specificity using peptide-stimulated LNC cultures derived from mice immunized with MOG 35-55 or Myhc-α 334-352 (data not shown). Second, to enumerate the frequencies of antigen-specific cells *ex vivo*, we used CD4 cells enriched from immunized mice. In the preliminary experiments, we confirmed that cells treated without NASE could be stained with dextramers, but the staining intensity was less distinct than in NASE-treated cells. Therefore, we treated the cells with NASE followed by tet or dext staining, and the cells positive for each were analyzed flow cytometrically. We noted that MOG 35-55 dextramers detected 3- to 5-fold higher numbers of MOG-reactive cells than MOG 35-55 tetramers, regardless of the subsets analyzed (CD4: 0.40% vs. 0.11%; CD25: 1. 74% vs. 0.57%; CD44: 1.61% vs. 0.35%). The staining was verified to be specific using PLP 139-151 tetramers or dextramers as controls, and their background staining was low (Figure 7A). We then performed staining on MNC harvested from CNS tissues from MOG 35-55-induced EAE in C57Bl/6 mice that had a score of 2 to 4. Cells were stained with MOG 35-55 tetramers or dextramers without exposing them to NASE. The analysis revealed that both MOG 35-55 tetramers and dextramers stained a fraction of cells, and the staining intensity was higher with dextramers than with tetramers (1.81% vs. 0.44%, as opposed to 0.42% vs. 0.23% for their corresponding controls; Figure 7B). Likewise, we analyzed the frequencies of Myhc-α-reactive cells *ex vivo *from immunized A/J mice using Myhc-α 334-352 dextramers or tetramers in different subsets, and the dext staining intensity was ~7 to 9-fold higher than that achieved with tetramers (CD4: 0.21% vs. 0.03%; CD25: 1.26% vs. 0.16%; CD44: 1.02% vs. 0.11%; Figure 8A). Although background staining with control reagents (RNase 43-56) was significantly lower than with Myhc-α tetramers or dextramers, the proportion of cells stained with RNase dextramers tended to be high. To enumerate the frequencies of Myhc-α-specific cells in hearts *ex vivo*, we used MNC harvested from hearts on day 21 postimmunization, and the cells were stained with Myhc-α 334-352 or RNase 43-56 (control) tetramers or dextramers. Figure 8B shows that while both Myhc-α tetramers and dextramers stained a fraction of CD4 cells, Myhc-α 334-352 dextramers detected ~5-fold higher number of Myhc-α-specific cells than with Myhc-α 334-352 tetramers (2.36% vs. 0.58%) and the background staining for corresponding control reagents was low (0.38% vs. 0.31%). Taken together, the data demonstrate that dextramers are useful tools to enumerate the precursor frequencies of antigen-specific cells *ex vivo *and their detection sensitivity is higher than that of tetramers.

## Discussion

Using three different murine autoimmune disease models, we have demonstrated that MHC class II dextramers permit *ex vivo *enumeration of self-reactive CD4 T cells with specificity. Tetramers, but not monomers or dimers, are useful in detecting CD4 T cells by FC [4,12,33]. In spite of this technical advancement, detection of autoreactive CD4 cells is not straightforward because of their rarity in the peripheral repertoires, and their detection often requires *in vitro *expansion and/or enrichment [33]. In this scenario, however, while antigen specificity of cells can still be confirmed, accurate estimation of their frequencies can be prone to error because of the need to activate cells prior to tetramer staining. Structurally, dextramers contain dextran backbones, which are polymers of glucose molecules attached through 1-6 and 1-3 linkages [15]. Each dextran molecule carries multiple moieties of SA to which biotinylated peptide-tethered MHC molecules can be assembled [15]. Therefore, MHC dextramers can yield MHC-peptide complexes of large molecular weight, allowing them to engage multiple TCRs - more than that could be achieved with tetramers. To address this hypothesis, we created MHC dextramers for three mouse IA alleles, IA^s^, IA^b ^and IA^k ^for PLP 139-151, MOG 35-55 and Myhc-α 334-352, respectively, and showed that the reagents detect corresponding antigen-sensitized cells with specificity and also to a higher sensitivity than conventional tetramers.

We optimized the conditions for dextramer staining using PLP-reactive cells generated from SJL mice immunized with PLP 139-151. Essentially, the conditions previously defined for conventional tetramers were also optimum for dextramers, and the staining was performed in growth medium supplemented with 2.5% FBS, pH 7.6 [5,11]. However, we noted some important advantages with dextramers, including enhanced sensitivity, shortened staining duration and the ability to create dextramers with a low amount of MHC/peptide complexes. We found that dextramers prepared at a 1:20 molar ratio of SA-dextran molecules to biotinylated MHC proteins were optimum to detect PLP-reactive cells, and their detection sensitivity was ~4-fold higher than that of tetramers. Importantly, the use of dextramers allowed us to capture antigen-specific cells by FC unambiguously as observed by an increase in their mean fluorescent intensities in relation to tetramers (Figure 1). This is an important consideration in flow cytometric analysis, especially when FITC-labeled reagents are used. In support of this notion, PLP 139-151 dextramers, but not tetramers, labeled with FITC detect nearly equivalent percentages of PLP-reactive cells similar to percentages obtained with PE- or APC-labeled dextramers. Furthermore, the enhanced detection of antigen-reactive cells could be achieved as early as 30 minutes postincubation with dextramers, whereas with tetramers, the staining intensity was only marginally improved even when extending the incubation time up to 3 hours. On a reaction basis, we calculated the amount of MHC protein required to prepare tetramers to be 2- and 4-fold higher than the amount of protein required to make dextramers labeled with PE and APC, respectively. The marginal reduction in the staining intensity obtained with FITC-dextramers was expected because signals generated from FITC are weaker than other fluorescent dyes. The fact that FITC-labeled dextramers, but not tetramers, bound PLP-reactive cells provides opportunities to include this dye in the FC-based multicolor analysis of antigen-specific T cell populations.

We believe that the higher detection sensitivity of dextramers is due to the presence of multiple SA moieties, allowing the formation of large clusters of MHC-peptide complexes, which can interact with multiple TCRs, thus enhancing the avidity of their interactions [15,33]. Conversely, each tetramer molecule contains a single SA moiety to which only up to four MHC-peptide complexes can bind, limiting the availability of MHC-peptide complexes to engage with multiple TCRs. As a result, tetramers might bind T cells bearing high- but not low-affinity TCRs. This appears to be the case because a proportion of tet^- ^cells sorted by FC could still be detected by dextramers (Figure 4A). Nevertheless, it should be noted that dextramers do not detect all antigen-sensitized cells, as indicated by the fraction of PLP-reactive cells previously negative for PLP dextramers that can become detectable after a second restimulation with PLP 139-151 (Figure 4B). Presently the significance of these cell populations is not known with respect to their TCR-affinities or functionalities, such as cytokine synthesis or encephalitogenicity. Alternatively, failure to detect all antigen-reactive cells with dextramers might be directly related to the density of TCRs expressed on the surface of T cells such that low TCR-expressed cells may escape detection. From a technical point of view, formation of stable MHC-peptide-TCR interactions with dextramers can help cells withstand harsh steps such as centrifugation and washing, thereby enhancing specificity by minimizing the background staining. Overall, the fact that dextramers permit quick determination of antigen-specific cells with enhanced sensitivity and because their creation requires less starting material, they have proven to be superior to conventional tetramers.

One of the limitations of class II tetramer binding is activation dependency [9-12]. Various strategies have been employed to enhance the detection sensitivity of class II tetramers by promoting interactions with multiple TCRs. These include antibody-mediated TCR clustering, MHC-peptide-coated liposomes and immunoglobulin molecules [14,34-36]. We had previously shown that, upon resting, antigen-sensitized cells that have expanded *in vitro *become undetectable with tetramers, but can be detected through NASE treatment prior to staining [11]. We asked whether the use of dextramers can overcome as the need for NASE treatment or activation. Expectedly, dextramers could bind rested cells in the absence of NASE treatment to a significantly greater proportion than that achieved with tetramers (Figure 5). The data suggest that activation dependency is not a critical factor for dextramer staining, and we verified this phenomenon further by *ex vivo *enumeration of PLP-specific cells in SJL mice immunized with PLP 139-151 (Figure 6). While, similar results were obtained with IA^b^/MOG 35-55 and IA^k^/Myhc-α 334-352 dextramers, NASE treatment added the advantage of enhancing their detection sensitivity as compared with tetramers (Figures 7 and 8). The data prove that MHC dextramers are useful tools for enumerating the frequencies of antigen-specific T cells *ex vivo *in various experimental systems.

## Conclusion

We have shown that dextramers are superior to tetramers in detecting autoreactive cells, which otherwise can be technically challenging. While activation dependency is less important for dextramer binding, detection sensitivity can be enhanced by NASE treatment without compromising the specificity. Although dextramers cannot detect all cells in spite of being antigen specific, their greater detection sensitivity makes them first-choice reagents over conventional tetramers. Additionally, the inherent variation in the detection abilities of these reagents is to be considered while interpreting the results because frequencies of antigen-specific cells identified by each reagent are expected to be different.

## Authors' contributions

CM designed and performed the experiments and wrote the manuscript. BU and AG participated in the design of the study and performed the experiments. CK performed the experiments. JR conceptualized the study, participated in the design and coordination and wrote the manuscript. All authors read and approved the final manuscript.
